# Immunotherapy Innovations in the Fight against Osteosarcoma: Emerging Strategies and Promising Progress

**DOI:** 10.3390/pharmaceutics16020251

**Published:** 2024-02-08

**Authors:** Shigao Cheng, Huiyuan Wang, Xuejia Kang, Hui Zhang

**Affiliations:** 1 Laboratory of Stem Cell and Tissue Engineering, Orthopedic Research Institute, Department of Orthopedics, West China Hospital, Sichuan University, Chengdu 610041, China; chengshigao@ldzxyy.com; 2 Department of Orthopedics, Hunan Loudi Central Hospital, Loudi 417000, China; 3 Shanghai Institute of Materia Medica, Chinese Academy of Sciences, Shanghai 200031, China; wanghuiyuan@simm.ac.cn; 4 Department of Drug Discovery and Development, Harrison College of Pharmacy, Auburn University, Auburn, AL 36849, USA; xzk0004@auburn.edu

**Keywords:** osteosarcoma, suppressive immune environment, nanoparticles, immunotherapy

## Abstract

Immunosuppressive elements within the tumor microenvironment are the primary drivers of tumorigenesis and malignant advancement. The presence, as well as the crosstalk between myeloid-derived suppressor cells (MDSCs), osteosarcoma-associated macrophages (OS-Ms), regulatory T cells (Tregs), and endothelial cells (ECs) with osteosarcoma cells cause the poor prognosis of OS. In addition, the consequent immunosuppressive factors favor the loss of treatment potential. Nanoparticles offer a means to dynamically and locally manipulate immuno-nanoparticles, which present a promising strategy for transforming OS-TME. Additionally, chimeric antigen receptor (CAR) technology is effective in combating OS. This review summarizes the essential mechanisms of immunosuppressive cells in the OS-TME and the current immune-associated strategies. The last part highlights the limitations of existing therapies and offers insights into future research directions.

## 1. Introduction

Osteosarcoma, the most prevalent malignant bone-related cancer in adolescents, poses a significant treatment challenge [1]. The survival rate for this condition over a five-year period ranges from 60% to 70%, and it is more frequently observed in males and individuals of African American descent [2]. Despite extensive research efforts, 5-year survival rates for osteosarcoma patients have remained around 20% in recent decades [3]. What is worse, the prognosis for patients with metastatic or relapsed osteosarcoma has remained bleak and stagnant over the past 30 years [4]. The current management strategy for primary OS is surgery plus neoadjuvant chemotherapy (preoperative treatments), and the survival rate has been discovered as 35–40% [5,6]. Rotation-plasty, a well-established method for knee tumor reconstruction, can yield functional and psychological outcomes equal or superior to endoprosthetic reconstruction [7,8]. However, it presents cosmetic challenges [7,9]. Axial skeleton sarcoma surgery is complex due to high local recurrence risk and frequent reconstruction complications [7]. About 30% of primary metastatic osteosarcoma patients and over 40% achieving complete remission can be long-term survivors [10]. Lung metastases in advanced OS patients decrease the 5-year survival rate to 5–10%; in this case, the adjuvant combination of chemotherapy and immunotherapy significantly enhances survival rates. Despite this progress, the immunotherapy of OS still requires in-depth investigation.

The tumor microenvironment (TME) is a complex system composed of not only cancer cells, but also various cell types, including immune cells such as myeloid-derived suppressor cells (MDSCs), osteosarcoma-macrophage (OS-M), and stromal cells like the cancer-associated fibroblast [11]. Among these, MDSCs are generated in the bone marrow, and in the context of cancer (tumor-bearing hosts), they further migrate to peripheral lymphoid organs and the tumor site [12] and exert immune suppressive activity [13], inhibiting the functions of cytotoxic T cells and natural killer cells [14]. Moreover, the tumor microenvironment in osteosarcoma (OS-TME) houses essential macrophage populations, which can be categorized into two main phenotypes: the classically activated macrophages, often referred to as Type-1 (OS-M1), and the alternative Type-2 macrophages (OS-M2) [15]. Within the OS-TME, the prevailing majority of macrophages primarily display the pro-tumor Type-2 phenotype, lacking anti-tumor functionalities [16]. Osteosarcoma-associated neutrophils also participate in the OS-TME [17]. Initially, neutrophils are responsible for immune attack; however, the OS educates the neutrophils to become tumor-promoting neutrophils [18]. Regulatory T lymphocytes (Tregs) in the immunosuppressive or cold OS-TME also matter, favoring OS progression and metastasis [19]. To effectively treat OS, it is essential to alter the OS-TME.

Traditional intervention approaches, such as surgical resection and chemotherapy, are primary strategies against OS. Cancer immunotherapy has surfaced as an important avenue for the improvement in OS-TME, benefiting the inhibition of metastasis and recurrence of OS [20]. For the therapy associated with the OS-M, we divided current immunotherapy for regulating the OS-M into two types: conventional immunotherapeutic and novel engineered cell therapy [21,22]. The conventional approaches encompass the inhibition of recruitment of tumor-promoting OS-M, the impairment of OS-M, and the recovery of OS-M phagocytosis function [23]. Nanotechnology is a significant platform for the therapeutics of modulating the OS-TME. Nanoparticles offer multiple advantages, including the ability to be intricately engineered for the robust and precise activation of T cells before their adoptive transfer [24]. Additionally, Nps can be used to provide the added functionality of immunotherapy [25,26]. Thus, nanoparticles present a promising solution to address the challenges associated with T cell therapy [27,28]. To enhance the accumulation of therapeutics at tumor sites, it is crucial for nanocarriers (NCs) to exhibit prolonged circulation, a characteristic achievable through surface modification with hydrophilic polymers like polyethylene glycol (PEGylation) [29]. Additionally, nanoparticles can also encapsulate agents with a size range of 50–100 nm and can effectively enter parenchymal hepatic cells, stimulating T cell activity [30]. Nanocarriers with a size less than 50 nm can penetrate the cellular barriers, promoting nanoparticle distribution into the bone lesion [31].

Another type of immunotherapy focus is on enhancing the natural ability of adaptive immune cells, particularly cytotoxic T lymphocytes (CTLs) [32]. Adoptive T cell therapy stands as a promising frontier in the realm of cancer treatment [33,34]. Clinical trials have provided compelling evidence of its potential in the context of both adult and pediatric populations, demonstrating the effectiveness of advanced immunotherapy in combatting bone cancer [23,35]. The impact of this therapy has been further underscored by the approval of three notable T cell-based therapeutics—Kymriah, Yescarta, and Breyanzi—by the US FDA [36,37,38]. However, though there has been considerable success in hematologic malignancy treatment, including B cell leukemia and lymphoma treatment, adoptive T cell therapy faces inherent challenges that limit its efficacy in the treatment of many solid tumors including OS [39]. The underlying reason includes the difficulty of T cell infiltration towards the solid tumor because of extensive surrounding stromal cells around OS [40]. To overcome this barrier, one of the approaches is to eliminate the stromal cells with other therapeutics, and nanoparticles can serve as efficient carriers for these therapeutics. Moreover, for future CAR T therapies, it is important to incorporate “on or off switches” that ensure CAR T efficacy in the tumor lesion [41]. Additionally, the serious side effects of CAR T such as cytokine release syndrome (CRS) and immune effector cell-associated neurotoxicity syndrome (ICANS) also hinder the success of CAR T [42,43]. Therefore, the combinations of anti-cytokine therapy or synergistic therapeutics to reduce the dose of CAR T will benefit CAR T cell application. In addition, it is reported that a third-generation GD2-CAR exerted effective recognition for the GD2+ sarcoma cell lines in vitro [44]. However, these GD2-CAR T cells were unsuccessful in an in vivo xenograft tumor model [44]. Similarly, it is significant to monitor the HER2 CAR T cell potency using a xenograft or validated model [45]. All finding indicated that the combinational strategies is significant. Another approach enhancing cancer specificity and clinical response is represented by a bispecific CAR T cell molecule [46,47,48].

In this review, we delve into the role of immunosuppressive cells within the OS-TME at first. Then we emphasize the pressing need for continued research and innovative strategies to address these challenges and expand the potential of T cell therapy in osteosarcoma. Additionally, we acknowledge the limitations that may hinder the viability of T cell therapy as a treatment option for this formidable bone malignancy. This perspective underscores recent advancements in preclinical research that leverage nanoparticles to augment the effectiveness of T cell therapy.

## 2. Immunosuppressive Osteosarcoma Microenvironment

Osteosarcoma is known for its high heterogenicity and low tumor immunogenicity. The extensive immune cell infiltration in the OS-TME leads to the formation of a niche for OS proliferation, metastasis, and resistance [17,49]. This immunosuppressive OS-TME is correlated with the presence of MDSCs (Myeloid-Derived Suppressor Cells), OS-Ms (osteosarcoma-associated macrophages), ECs (endothelial cells), and Tregs (regulatory T lymphocytes) [50]. Among them, MDSCs are a heterogeneous population of immune cells that play a crucial role in suppressing the anti-tumor immune response [51]. OS-M refers to tumor promoting macrophages, which, in general, contribute to tumor progression via promoting angiogenesis, tissue remodeling, and immunosuppression. ECs (endothelial cells) are responsible for the supply of nutrients [52,53]. Regulatory T lymphocytes (Tregs) is beneficial for the immune surveillance [19].

### 2.1. MDSC

As per the brief discussion on MDSCs in the introduction, MDSC is significant component in the OS-TME. Even in the initial stage, MDSC contributes to the pathogenesis of OS through several mechanisms [14]. Firstly, MDSCs hinder T cell migration and reduce T cell viability, making T cell access to the OS more difficult [54]. Furthermore, MDSCs alter T cell fitness by the production of immune-inhibitory molecules like nitric oxide (NO), reactive oxygen species (ROS), and reactive nitrogen species (RNS). Additionally, MDSC reduces T cell-mediated immune responses via the consumption of L-arginine [55]. Lastly, MDSCs consume vital metabolites necessary for T lymphocyte fitness, further compromising the immune response [56]. Additionally, MDSCs can further migrate to peripheral lymphoid organs, resulting in antigen-specific T cell tolerance, and contributing to the metastasis of OS [12]. Firstly, MDSCs play a role in promoting tumor angiogenesis through the secretion of factors like vascular endothelial growth factor (VEGF) and matrix metalloproteinase 9 (MMP9) [57]. Both of them support the growth of micro-vessels within the tumor and aid the tumor’s expansion [58]. MDSCs also secrete elevated levels of transforming growth factor-beta (TGF-β) and hepatocyte growth factor (HGF) to benefit the growth of OS in other distant organs [59]. The existing TGF-β and HGF induce epithelial–mesenchymal transition (EMT), a process that enhances the tumor’s ability to invade and metastasize [60]. In the metastatic niche, MDSCs secrete a molecule called versican to contribute to the establishment of metastatic tumor growth [61].

### 2.2. OS-M

Besides MDSCs, in the OS-TME, OS-M functions as a mutineer [62]. In the initial harsh OS-TME characterized by factors such as hypoxia, low pH, elevated glutathione (GSH) levels, and dysregulated kinase systems [63,64], OS cancer cells educate macrophages to adopt tumor-proliferation-supportive roles. Then, as a feedback, the infiltration of OS-M further aggravates the PD-L1 expression in OS, negatively impacting the cytotoxicity of T cells [65,66]. Furthermore, the hypoxic environment in OS-TME promotes tumor angiogenesis [67,68]. In addition, tumor cells also release signals like IL12 and IL4, along with hypoxia-inducible factors HIF-1α and HIF-2α, to maintain OS-M education and support the dysfunction of DCs [69]. OS-M elevates the levels of vascular endothelial growth factor as well as matrix metalloprotease 9 [70]. This facilitates angiogenesis and the formation of a pre-metastatic niche, demonstrating a strong association with osteosarcoma metastasis [71].

### 2.3. Endothelial Cell

Endothelial cells (ECs) play a role in promoting the acquisition of tumor cell properties, including cell growth, invasion, metastasis, and chemoresistance [72,73]. EC proliferation is associated with nutrient supplies for the OS-TME [74]. It is identified that cyclin-dependent kinase 2 and 5 (Cdk2, Cdk5) serve as key mediators of neo-angiogenesis [75,76]. Additionally, a specific signal named Yin Yang 1 (YY1) protein from osteosarcoma (SaOS) cells plays a crucial role in driving the proliferation of human aortic endothelial cells (HAECs) [76]. In addition to resident endothelial cells, there are circulating ECs. Elevated levels of circulating endothelial cells (CECs) have been found in the peripheral blood of OS patients compared to control groups [77,78]. On the contrary, circulating endothelial progenitor cells (CEPs) are cells derived from the bone, specifically contributing to tumor-associated vasculogenic effects [79]. Additionally, ECs function as both modulators and effectors in the context of OS, contributing to the acceleration of OS exacerbation through the release of von Willebrand factor (VWF) [80].

### 2.4. Treg

Treg cells represent a dynamic subset of CD4+ T lymphocytes that regulate both normal and aberrant immune system responses [81,82]. Tregs in the TME play critical roles in enabling tumor cells to evade immune surveillance [83]. Some important molecules associated with Tregs will be discerned in this part. For instance, CD39, an ectonucleotidase elevated on Treg cell surfaces, facilitates immunosuppression [84,85]. CD39 converts adenosine triphosphate to adenosine; the subsequent biological binding of adenosine to the A2A receptors (A2AR) and/or A2B receptors (A2BR) has a negative impact on the functions of natural killer and dendritic cells in the TME [85,86,87]. What is worse, adenosine potentiates the expansion of tumor promoting cells, including MDSCs and OS-M2 [87]. Additionally, Tregs secrete perforin and granzymes affecting effector T cells [88,89]. The neuropilin-1 (Nrp1) semaphorin-4a (Sema4a) axis is a newly found factor associated with Tregs in the TME [90]. In addition, CTLA-4 on Tregs causes the direct suppression of the APC function of DCs and hampers the abilities of effector T cells [91]. Thus, the manipulation of Treg function through therapeutic interventions has become a promising strategy.

### 2.5. OS-Ns

OS-Ns refers to osteosarcoma-related neutrophils [92,93]. OS-Ns has phenotypic heterogeneity and functional versatility. In osteosarcoma, research on TANs is still in its early stages. The lifespan of OS-Ns is longer than that of circulating neutrophils [94,95]. Liu et al. used a meta-analysis to examine the possible correlation between matrix metalloproteinases -9 (MMP-9) mediated by OS-N and a poor prognosis for patients [94,95]. The higher the level of matrix MMP-9 expression, the higher the poor-prognosis risk of patients with OS [94]. Of note, this study faced challenges from another researcher, and thus more studies are essential to validate the prognostic value of MMP9 in OS [94,96]. Neutrophil extracellular traps are web-like chromatin structures formed by the granule proteins and chromatin generated by OS-Ns, which contribute to metastasis, and the underlying mechanism is associated with the DNA receptor coiled-coil domain which includes protein 25 [97]. Another significant chromatin that forms OS-N extracellular traps is peptidylarginine deiminase 4 which overexpressed on OS [98]. Furthermore, the infiltration of neutrophils promotes the translation of hypoxia-related genes, resulting in an increasingly hypoxic microenvironment [99]. A hypoxic TME is not beneficial for the efficacy of anti-cancer therapy [100,101]. It is also deserve to mention that the predominant OS-N in the OS lesion significantly increases the immune escape evasion of OS cells [99].

## 3. Interactions of Tumor Promoting Cells in the OS-TME

Osteoimmunology is a new term used to describe the immune TME in bone-related disease. The abovementioned immune cells facilitate the evasion of immune attack via immunoediting or other escape mechanisms [102,103]. The OS cell itself is able to downregulate human immunity or achieve immune escape via PDL1/L2 [104], B7-H3 [105], HHLA2 [106], or MHC class II [52,107]. In addition, OS cells release abundant VEGF that interacts with VEGFRs in the endothelial cells (ECs), benefiting angiogenesis and facilitating OS nutrient supply [108].

**Figure 1:**
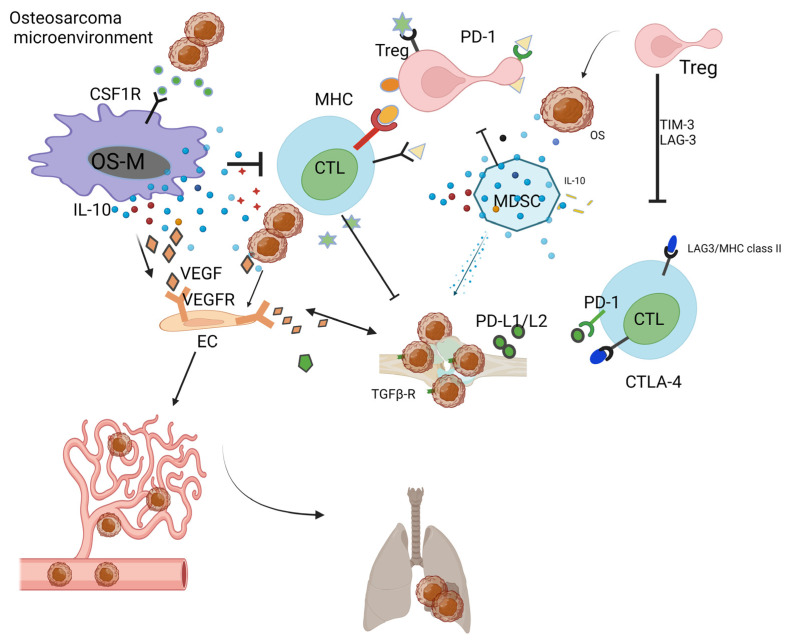
Immunosuppressive OS-TME: Immune-related cells including OS-M, CTL, Treg, MDSC, and OS-N closely work with each other, facilitating the formation of immunosuppressive OS-TME that benefits the proliferation of OS. The MDSC, OS-M, and Treg benefit angiogenesis via VEGF pathways. The OS-TME favors the cancer metastasis process from the primary bone site to the distant lung site. Created with biorender.com, access on 25 October 2023.

OS cells release TGF-beta to increase the ratio of Treg in the OS-TME [17]. MDSCs release IL-10 and TNF-alpha, which decrease the activity of cytotoxic T lymphocytes (CTLs) [109]. In the OS-TME, after neo-adjuvant chemotherapy, elevated cytotoxic lymphocyte TILs are associated with a decrease in MDSCs [110]. OS-Ms generally exert pro-tumoral function and show a high correlation with OS aggressiveness and poor prognosis [110,111]. In the OS-TME, OS-M also benefits the formation of neo-vessels via the interaction of the VEGF/VEGF receptor [52]. CTL is responsible for the killing of OS; however, the development of immune surveillance pathways, including the PD-L1/L2-PD1 andCTLA-4 [112], T cell immunoglobulin and mucin-domain-containing-3 (TIM-3) as well as lymphocyte activation gene-3 (LAG-3) [113], ensures the immune escape of OS [62]. Recent preclinical studies have evidenced the TME-promoting role for Tregs in OS [114]. Yoshida. et al. reported that the decrease in Tregs is paralleled by the increase in TILs in the OS-TME [115]. Tregs negatively impact the cytotoxic activity of T cells via TIM-3 as well as LAG-3 [113]. Immunoglobulin and tyrosine-based inhibitory motif (ITIM) domain (TIGIT) on T cells have surfaced as immune targets [116]. All cells actively interact with each other and form an immunosuppressive OS-TME (Figure 1).

## 4. Conventional Nanoparticle-Based Therapy and Engineered Immunotherapy for OS-M

OS-dominated tumor-promoting macrophages possess characteristics suppressing immunity and promoting tumor development [117,118]. Thus, it is of importance to treat OS-M. Given the massive investigations associated with macrophages, we have introducted nanoparticles for OS-M in one section and will discuss the applications of nanoparticles for other immune cells in OS-TME later. Traditional approaches for the OS-M include three approaches. Firstly, reducing the source of OS-M using nanoparticles containing inhibitors of recruitment pathways such as CSF1 [119], CCL2 [120], CCL5 [121], and CXCL12 [122] pathways. By inhibiting these pathways, monocyte recruitment into osteosarcoma lesions can be decreased, consequently reducing the differentiation of resident OS-Ms. Secondly, nanoparticles carry therapeutics that interfere with the function of tumor-promoting OS-Ms. For instance, in one study, when treating OS with graphene oxide with PEG modification, pro-tumoral OS-M2 was suppressed, consequently resulting in the regression of bone cancer [123]. Thirdly, certain nanoparticles, either alone or conjugated with antibodies, have demonstrated the ability to restore macrophage phagocytosis. For instance, siRPa-CD47 blockage can be achieved using nanoparticle-based approaches [124]. Other nanoparticles potentially impacting MCHII and PD1/PDL1 pathways can also recover phagocytic abilities [20,125,126]. Furthermore, the combination of CD47 mAb and ferumoxytol-loaded nanostructures can enhance the anti-tumor OS/M1 ratio [127]. CTLA-4 (aka CD152) is regarded as overexpressed in the OS-TME [128]. The synergistic application of CTLA-4 inhibitors with nanoparticles provides insights for OS treatment [129]. In addition, PD-1/PD-L1 is reported to negatively correlate to the survival rate, and this pathway has emerged as a target in osteosarcoma [130]. The blockade of PD-1 using pembrolizumab leads to the inhibition of osteosarcoma [131,132]. Because macrophage is a type of immune cell, so the therapy targeting macrophage can be regarded as conventional immunotherapy.

**Figure 2:**
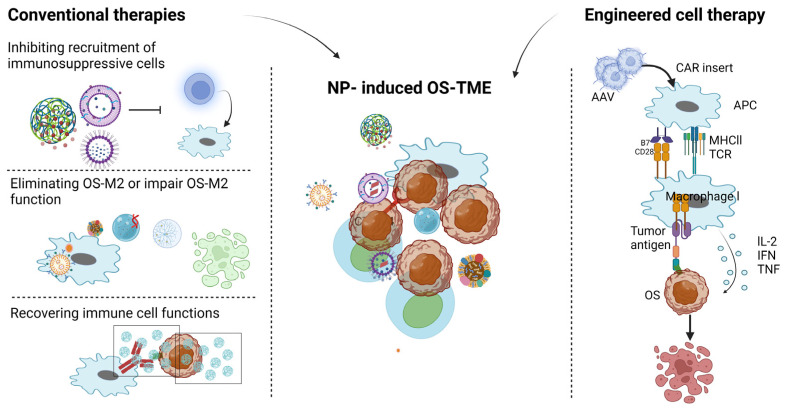
Illustration of TAM therapies: traditional approaches and engineering cell approaches. Traditional therapies involve established methods inhibiting OS-M recruitment and function. These approaches aim to eliminate cancer cells and modulate the immune response. The corresponding elements in the figure highlight the key components and interactions involved in traditional approaches.

Additionally, CAR-M technology presents a novel therapeutic approach for modifying tumor-promoting macrophages into anti-tumor macrophages, thereby enabling macrophages to recognize combat cancer [133]. Because although conventional therapies are promising in modulating OS-M, the effects cannot be maintained for a long time due toa defense mechanism developed by OS [134]. Thus we need immune cell that has memory. CAR-based therapy has been regarded as “living drug” [135]. Chimeric antigen receptor macrophage (CAR-M) cells has gained prominence in the solid tumor field [136]. However, it need to point out, currently, there is no promising progress for CAR-M in OS treatments. Still, we would like to introduce CAR-M and hopefully it will provide insights for future researchers. In CAR-M therapy, isolated patient immune cells (monocytes) are engineered to express chimeric antigen receptors and then are infused back to the patient [137]. Consequently, the CAR modification can help macrophages to discern cancer cells, making modified macrophages a promising tool in the fight against osteosarcoma [138,139]. The initial efforts to introduce CARs into macrophages were pioneered by Biglari and colleagues in 2006, in which a chimeric CD64 molecule was developed, comprising a single-chain variable fragment (scFv) designed to target the human carcinoembryonic antigen (CEA), along with the transmembrane and cytoplasmic domains of human CD64, resulting a significant reduction in the tumor growth rate in a xenotransplantation model [140]. Additionally, the CAR-M technology presents a novel therapeutic approach for enhancing the macrophages’ ability to phagocytose cancer cells and present antigens, thereby enabling macrophages against cancer [125]. Engineered CAR-Ms have resulted in increase of anti-tumor cytokines, such as IL6, and chemokines, such as CXCL18, in the OS-TME [141,142]. The production of these beneficial cytokines fosters a shift from a cold OS-TME towards a hot OS-TME [143]. Nothing is perfect, it also should pay attention to the levels of IL6, massive IL6 can cause cytokine release syndrome [144]. Alternatively, other investigators used computational modeling tools to anticipate the performance of a newly designed CAR molecule capable of facilitating bispecific antibodies (Figure 2).

## 5. Advanced Immunotherapy—Chimeric Antigen Receptor (CAR)-T Cell Therapy

### 5.1. Chimeric Antigen Receptor (CAR) T Cell Immunotherapy

In addition to the conventional NP-associated therapies, the successful implementation of chimeric antigen receptor (CAR) T cell immunotherapy in the treatment of hematological malignancies proves to be a possible strategy worth exploring in the context of OS [145]. CAR-modified T cells utilizing the immune system’s potent cytotoxic mechanisms against tumor cancer cells have shown promising progress [146]. Nevertheless, the development of successful CAR T cell therapies for solid tumors encounters challenges. [147,148] Thus, combinational therapy has been considered as a more promising approach. It is reported that OS prominently expresses the GD2 antigen, which has been identified as a viable immunotherapeutic target [149]. In addition, researchers have evaluated the potential of CAR-modified T cells targeting GD2 to induce cytotoxicity against OS tumor cells [150,151]. It is demonstrated that GD2 CAR-modified T cells exhibited high efficacy in inducing cell death in OS tumor cells [151]. However, the OS cells gradually developed the ability to escape the attack of GD2 CAR-modified T cells via PD-L1 expression in OS cells [152]. The PDL1–PD1 interaction gives the wrong signal to CAR T cells, stopping the activity of CAR T cells and finally inducing CAR T cell apoptosis [153]. A cyclic peptide, CGRRAGGSC (using the single-letter amino acid code), displayed on an IL-11 mimic phage exhibited specific binding to immobilized IL-11Rα [154,155]. 

Importantly, IL-11Rα has been recognized as a target for OS molecular focus in CAR T cell studies [152]. In a study conducted by Moonat, Hatel et al., engineered CAR T cells, using IL-11Rα as the tumor-specific immunoreceptor, exhibited enhanced cytotoxicity against multiple OS cell lines [156]. Moreover, there have been investigations into the use of γδ T cells in the context of osteosarcoma [157,158]. Interestingly, zoledronic acid enhances the susceptibility of rhabdomyosarcoma cells to destruction by human γδ T cells [159]. Additionally, HER2-specific CAR T therapy has been explored in mouse models of osteosarcoma. phA2 CAR T cells effectively target and eliminate EphA2-positive osteosarcoma and Ewing’s sarcoma cells in vitro and induce complete tumor regression in subcutaneous xenograft models [160]. In vivo, direct injection into established tumors extends survival, while systemic delivery requires a higher cell dose. In murine osteosarcoma, a strategy involves combining CTLA-4 blockade either with tumor lysate-pulsed dendritic cells (DCs) or PD-L1 blockade therapeutics [160]. Building on this, studies have confirmed that the co-administration of lenalidomide with EGFR vIII-CAR T cells enhances F-actin polymerization, CAR T cell infiltration, and cytotoxicity at the tumor site in a murine glioblastoma model, ultimately extending the survival of treated mice [136]. CD28-based (NCT00902044) and CD28-CD3z-OX40 CAR T cell therapy (NCT01953900) have been used in sarcoma patients in clinical trials. Moreover, a trial investigating the combination of CAR T cells with low-dose chemotherapeutic agents is still ongoing (NCT04433221). More information associate with CART please refer to Table 1. Because there is no commercial product specific for OS, thus we summarized the progression of CART in liquid cancer. 

The successful infiltration of modified T cells is significant and nanoparticles can be useful in tracking CAR T. For instance, iron oxide nanoparticles offer a clinically applicable method for tagging CAR T cells, facilitating the noninvasive tracking of these labeled cells through magnetic particle imaging (MPI), photoacoustic imaging (PAT), and MRI. This enables the identification of their distribution within the body [161]. In this study, T lymphocytes labeled with the Fe2O3 nanoparticles were found in osteosarcoma [161]. This study explores the monitoring of CAR T cells with ferumoxytol, opening up the possibility of detecting CAR T cells in solid tumors [161]. 

**Table 1:** The examples of successful CAR T therapy in liquid cancer and solid cancer.

Targets	Disease	Outcomes	Product/Pre-Clinical Trail/Clinical Trails
CD19	Acute lymphoma leukemia	Success in pre-clinical study	[36]
CD19	Acute lymphoma leukemia	Success in pre-clinical study	[37]
CD19	Relapsed/Refractory B cell precursor ALL	Success	Tisagenlecleucel/Kymariah
CD19	Relapsed/Refractory large B cell lymphoma	Success	Axicabtagene ciloleucel/Kymariah
CD19	Relapsed/Refractory follicular lymphoma (FL)	Success (May 2022, FDA-approved)	Axicabtagene ciloleucel/Kymariah
BCMA	Relapsed/Refractory multiple myeloma	Success (February 2022, FDA-approved)	Ciltacabtagene autoleucel/Carvykti
BCMA	Relapsed/Refractory multiple myeloma	Success (March 2021, FDA-approved)	Idecabtagenevicleucel/Abecma
CD19	Relapsed/Refractory large B cell lymphoma	Success (February 2021, FDA-approved)	Lisocabtagene autoleucel/Breyanzi
CD19	Relapsed/Refractory B cell lymphoma	Success (October 2017, FDA-approved)	Axicabtagene ciloleucel/Yescarta [162]
CD19	Relapsed/Refractory follicular lymphoma (FL)	Success (March 2021, FDA-approved)	Axicabtagene ciloleucel/Yescarta
IL13R	Glioblastoma	Success in preclinical trials	[163]
Epidermal growth factor receptor variant III (EGFRvIII)	Recurrent Glioblastoma	Success in human patient	[164]

### 5.2. Inhibition of the Interaction between Immunosuppressive Cells to Improve CAR T Function

MDSC and OS-M have significant negative implications for the immune response in osteosarcoma [110]. Hence, modifying the conditions within the tumor microenvironment represents another potential approach to enhance the effectiveness of CAR T cell therapy [165]. In a clinical trial involving third-generation CD19 CAR T cell therapy, low levels of monocytic MDSCs (M-MDSCs) were observed. M-MDSCs were associated with a positive treatment response in patients with lymphoma and leukemia [166]. This suggests that a lower presence of M-MDSCs in the patient’s immune system may be correlated with better outcomes when using CAR T cell therapy to treat these types of cancers [167]. The presence of MDSCs, which can suppress the immune response, is a significant factor to consider when evaluating the success of immunotherapies like CAR T cell therapy [165]. Strategies to mitigate the effects of MDSCs and enhance the immune response has attracted investigators’ attention. The removal of MDSCs using an anti-Gr-1 antibody demonstrated the enhanced effectiveness of CAR T cell therapy in mouse models [168,169]. Moreover, CAR T cells designed to target the tumor vasculature via VEGFR-2 were successful in decreasing MDSCs within the TME [170]. Notably, MDSCs also expressed VEGFR-2, the same target as the CAR T cells, indicating a potential mechanism for their reduction [170]. When CAR T cells were combined with substances like polyinosinic-polycytidylic acid (poly I:C), all-trans retinoic acid (ATRA) [166], and gemtuzumab ozogamicin (GO) [171], enhanced anti-tumor effectiveness was observed. The suppressive activity of MDSCs was associated with an increase in therapeutic efficacy [172,173]. TAMs secrete immunosuppressive cytokines such as transforming growth factor-beta (TGF-β) and interleukin-10 (IL-10) [174]. These cytokines support the differentiation and expansion of Tregs while inhibiting the activity of effector T cells [174]. TAM-derived TGF-β, in particular, has been implicated in promoting Treg development and function [59]. Additionally, Tregs can reshape the profiles of immune cells within the tumor, suppressing IFN-γ secretion by CD8+ T cells and promoting the development of OS-M through sterol regulatory element-binding protein 1 (SREBP1)-mediated fatty acid synthesis [175,176]. The inhibition of these cytokines transforms the tumor microenvironment and benefits the prevention of cancer cell immune escape, thus improving CAR T function [149].

### 5.3. Limitations of Engineered Cell Therapy

Another challenge is the infiltration of CAR immune cells. To improve their infiltration into human tumors, we can try the following method: develop approaches to modulate the tumor microenvironment in a way that facilitates CAR T/M infiltration, which involves altering the levels of immunosuppressive factors or increasing the presence of tumor-specific chemo-attractants.

It is important to consider how chemokine signaling pathways can be integrated into the treatment strategies for osteosarcoma, especially in combination with immunotherapies. An important pathway for the CAR targeting these pathways may be the modulation of the tumor microenvironment, reduction in immunosuppression, and improvement in the effectiveness of immunotherapeutic approaches in osteosarcoma.

In addition, designing CAR T/M cells with improved homing mechanisms to recognize and migrate to specific tumor sites more efficiently may represent another strategy. As we know, the CAR scFv recognizes and binds to the specific tumor-associated antigen (or other targeted antigen) on the surface of cancer cells or infected cells. And, the intracellular domains of the CAR contain signaling elements, typically derived from co-stimulatory molecules (e.g., CD28 and 4-1BB) and the CD3ζ chain, for which signaling domains activate downstream signaling cascades upon antigen binding so we can further develop new co-stimulatory molecules. Moreover, we can fine-tune the design of CARs used in CAR-M therapy to ensure they are well-suited for navigating the complex human tumor microenvironment.

Additionally, to improve the effects of CAR T/M, combinatorial therapies are good strategy. For instance, CAR-M therapy can be combined with other treatments, such as checkpoint inhibitors, to help overcome immunosuppression in the tumor microenvironment and promote CAR-M infiltration. To further improve infiltration, a drug delivery strategy can also be considered. Research can focus on exploring local delivery methods to directly introduce CAR-M cells into or near tumor sites, potentially bypassing some of the obstacles posed by the complex tumor microenvironment.

## 6. Other Nanoparticles Affecting OS-N and Treg

Due to the pro-tumor impact of MDSCs in the OS-TME- [74], the blocking, reprogramming, or elimination of MDSCs has been surfaced as an avenue for transforming the OS-TME. For instance, to decrease the ratio of MDSCs in the TME, a nanoparticle named HA/ZIF-8@Gem/D-1-MT NP was designed to treat OS [22]. The sufficient encapsulation of the chemotherapeutic agent gemcitabine in the nanoparticle exerts cytotoxic activity, and, in another aspect, the incorporated IDO inhibitor 1-methyl-DL-tryptophan (1-MT) can suppress IDO [22]. MDSC is an inflammatory cell in the OS-TME. Nano-based efforts reduce tumor-related inflammation and can modulate the immune response in the OS-TME. Indocyanine green-encapsulated calcium phosphosilicate nanoparticles (ICG-CPSNPs) were utilized to impact the OS-TME. Via the mitigation of inflammation-MDSCs, the use of ICG-CPSNPs generates PDT effects inhibiting OS [177].

The reduced population of T regulatory cells (Tregs) within the TME can enhance the effectiveness of a new nanoparticle hyperthermia therapy [178]. However, keywords such as “nanoparticle for treg cell in osteosarcoma” and “inhibition of function of regulatory T cells in osteosarcoma using nanoparticles” provide nearly zero results, suggesting the absence of nanoparticles using Treg as a direct target in the OS-TME. The HA/ZIF-8@Gem/D-1-MT NPs discussed above not only suppressed MDSC but also reduced the regulatory T cells [22]. Hongbin Fan and his colleagues unraveled the diversity and immunosuppressive role of regulatory T cells in osteosarcoma via the analysis of single-cell RNA transcriptome data [179].

## 7. Other Nanoparticle Effects

Nanoparticles offer significant potential for delivering a range of anti-osteosarcoma agents, and ongoing research in this area promises novel insights and strategies for combatting osteosarcoma through immunotherapy [180]. Firstly, as we discussed nanoparticles (Nps) can be used for the temporal and spatial regulation or modulation of Tregs, TAMs, and MDSCs in the OS-TME. Nps offers a strategy to transform “cold” OS-TME into “hot” OS-TME, enhancing the immune response within the tumor microenvironment [181]. Secondly, the nanoparticle-based therapy regenerates the dying OS and causes immunogenic cell death (ICD) effects. Moreover, nanoparticles have favorable optical and magnetic attributes, making them suitable for combination with other therapeutics, including photodynamic therapy (PDT) [182]. Thirdly, nanomaterials improve the efficacy of CAR T or CAR-M in OS treatment [125].

### 7.1. NP-Induced ICD

Photodynamic therapy (PDT) can cause cancer cell apoptosis in osteosarcoma (OS) via the production of reactive oxygen species (ROS) [5,6]. Nanocarrier encapsulated photosensitizers (PSs) increase PDT-induced tumor cell death. More importantly, the balance between apoptosis and autophagy is of significance in the rescuing pathway against cell death, and a lower ratio of autophagy leads to backfiring effects, i.e., triggering the anti-tumorigenic effects in the PDT-treated TME [9]. However, inhibiting autophagy increases calreticulin (CRT) induced by ROS, suggesting elevated immunogenic cell death (ICD) [10,11]. Given the controversial findings, additional in-depth PDT studies on OS are still required. In a study, researchers designed a bovine serum albumin-zinc phthalocyanine (BSA-ZnPc; BZ) to assess the above findings. In this study, the OS tumor was resected at first, followed by BSA-ZnPc treatments. With combinations, the autophagy protein beclin, ATG5, and LC3II increased; however, the contrary protein p62 decreased, suggesting an increased level of autophagy. Furthermore, when cells were treated together with autophagy inhibitor, 3-MA, PD-L1 expression in cancer cells increased, indicating the correlation of decreased PDL-1 and decreased autophagy levels [183]. Moreover, gold nanoparticles encapsulated within tumor cell membranes offer the potential for precise drug delivery to specific sites, controlled drug release, and effective phototherapy [184]. As an example, researchers developed a mitochondria-targeted nano micelle, called OPDEA-PDCA, with the ability to induce mitochondrial oxidative stress and trigger pyroptosis in osteosarcoma cells [185]. In this study, mitochondria were specifically targeted. Additionally, the authors incorporated modified dichloroacetate (DCA) to inhibit pyruvate dehydrogenase kinase 1 (PDHK1), thereby inducing mitochondrial oxidative stress, causing pyroptosis, and further facilitating ICD [185]. Several studies have demonstrated that when osteosarcoma cells undergo cell death, dying OS cells become immunogenic and subsequently boost the immune response in the human body [186,187]. The combinational treatments initiated immunogenic cell death (ICD), which enhanced the macrophage’s innate immune response [188]. In animal models of metastatic osteosarcoma, the combined blockades of CTLA-4 and PD-L1 completely eliminated the bone carcinoma. Moreover, the addition of tumor lysate-pulsed dendritic cells (DCs) exerted a strong immune response in murine osteosarcomas [189].

### 7.2. NP-Induced Cold OS-TME to Hot OS-TME Transformation

The concept of cold OS-TME indicates the immunosuppressive OS-TME, where the immune reaction is dominant. To treat the OS, we need to heat the OS-TME. As mentioned, the MDSC inhibits the immune system’s response. Thus, the depletion of immune-inhibitory cells, such as MDSCs, benefits the hot OS-TME construction [190]. Additionally, combinations of inhibitory cytokines like IL-4 and TGF-β can contribute to a comprehensive approach to enhance the antitumor immune response [136]. A good example is gemcitabine (Gem) plus docetaxel or sirolimus. The underlying anti-cancer mechanism is associated with the impairment in DNA replication in cancer cells. The combinations exert excellent effects towards OS treatment; however, current therapeutic outcomes remain an unsolved challenge. To solve this dilemma, an innovative nanoparticle (HA/ZIF-8@Gem/D-1-MT NPs) has been designed to efficiently encapsulate the chemotherapeutic drug gemcitabine and an IDO inhibitor, 1-methyl-DL-tryptophan (1-MT) [22], leading to positive therapeutic effects and a synergistic anti-tumor immune response in OS-TME [22]. Zoledronic acid, a third-generation bisphosphonate, triggers apoptosis in tumor cells and inhibits angiogenesis and metastasis in vivo. This effect is achieved by arresting the S phase and activating DNA damage in cancer [191]. Indoleamine 2,3-dioxygenase (IDO) is an enzyme responsible for converting tryptophan into inhibitory metabolites that hinder T cell proliferation, creating an environment for tumor cells to evade the immune system [192]. In osteosarcoma, the combination of IDO inhibitors and platinum (IV) prodrugs acts synergistically to reverse low immune responses [193]. This combined approach enhances the efficacy of cancer chemotherapy and immunotherapy for osteosarcoma [193]. Moreover, a metal–organic framework enhances chemo-immunotherapy for osteosarcoma by inhibiting MDSCs and indoleamine 2,3-dioxygenase [22].

The activation of stimulator of interferon genes (STINGs) in the TME suggests the production of anti-tumor cytokines and interferons; thus, the level of STING is an indicator of the cold TME and hot TME [194,195]. Activating the STING pathway with NPs could enhance the anti-OS effect [196]. Additionally, radiotherapy can activate the cGAS-STING pathway by inducing DNA breaks [197]. The combination of hafnium oxide NP with radiotherapy can augment the efficacy of radiotherapy in patients with soft tissue sarcoma [197]. It is reported that Pt(IV)–C12 and IDOi (NLG919) not only hindered tryptophan metabolism in cancer cells but also caused DNA damage and triggered the STING pathway [197]. The impact of NP-Pt-IDOi on inhibiting cancer cells was assessed using various osteosarcoma cell lines in laboratory settings [186] docetaxel (DTX) and alendronate (ALD) were incorporated into chitosan-conjugated PLGA NPs to against OS [198,199]. These NPs, prepared by the nanoprecipitation technique, are found within the tumor targeting range (~200 nm) and display an effective positive charge (20 mV) capable of increasing cellular uptake efficiency [198,199].

### 7.3. NP-Improved TME for Combating Immune-Based Therapy Resistance in OS

Why Is There Resistance to ICD Effects in OS-TME? First, ABCB1 lowers the intracellular accumulation of ICD reagents such as doxorubicin, preventing DOX from reaching a concentration sufficient to induce ICD [187]. Additionally, ABCB1 increases the cell response towards ER stress and therefore impairs the magnitude of ICD [200,201] Finally, the resistant genes in OS cells hinder the functions of anti-cancer factors-calreticulin developed with the ICD effects triggered by immunotherapeutic ingredients [202]. In addition to ABCB1, OS cells release soluble factors, including bone-derived molecules such as transforming growth factor-beta (TGF-βs), being notable for inhibiting T cell function [50,203]. MDSCs and derived indoleamine 2,3-dioxygenase (IDO) also contribute to the OS-TIME [22,204,205]. TAM-induced vascular endothelial growth factor (VEGF) is an emerging factor in the OS-TIME [206]. Thus, to recover the immunity of cells, a protective microenvironment for immunotherapy for OS is important. Nanoparticles -Induced OS-TME is effective in transforming the TME. Np incorporating both albendazole (ABZ) and doxorubicin were designed, and the ABZ component of these nanoparticles demonstrated effective suppression of the expression of HIF-1α and its downstream protein VEGF [207]. In this study, the combination of ABZ with DOX drastically increases intracellular reactive oxygen species (ROS) and thus elevates tumor cell apoptosis. More significantly, ABZ effectively suppresses the expression of its downstream vascular endothelial growth factor (VEGF) via downregulating the levels of hypoxia-inducible factor-1α (HIF-1α) [207]. It is worth to mention that MDSCs contribute to bone resorption by activating osteoclasts in an HIF-1α-dependent manner [14]. HIF-1α further promotes the release of soluble factors, including basic fibroblast growth factor (bFGF) and matrix metalloproteinase-9 (MMP-9), favoring OS cell invasion [200]. Additionally, nanoparticles can be engineered to modulate the immune response, potentially overcoming immunosuppressive factors present in the OS-TME [132,165,208]. Nanoparticles can enhance the function of T cell therapeutics and promote a more robust anti-tumor immune response [209]. Because of the lower level of Her2 in the OS, the immunotherapy for Her2 alone is not effective in OS treatment, and graphene oxide nanoparticles conjugated with anti-Her2 Trastuzumab dramatically reduce OS growth [210]. The coating of OS cell membrane can increase the uptake of therapeutics in OS cells. Nanoparticles coated with a K7M2 membrane (derived from a murine (mouse) model of osteosarcoma) carrying ginsenoside Rh2 and alendronate increase the antitumor activity of TILs and decrease immunosuppressive Treg cells [211]. Curcumin-loaded nanoparticles stimulate autophagy, increase DC infiltration, and show synergistic effects with anti-PD-1/PD-L1pathway inhibitors [212]. Telomerase-specific oncolytic adenovirus OBP-502 in combination with anti-PD-1 increases efficacy by inducing autophagy, restoring ICD, and upregulating PD-L1 [213]. To sum up, in the NP-induced OS-TME, tumor-promoting factors such as ROS, VEGF, HIF-A, Bfgf, and MMP-9 can be decreased.

### 7.4. Engineering Cell Approaches

The cutting-edge field of engineering cell therapies represents a novel and promising avenue for tackling osteosarcoma-associated macrophages. This approach involves manipulating or modifying immune cells, such as macrophages, to enhance their anti-tumor activity. Genetic engineering, immunotherapy, and cell-based therapies fall under this category. The figure details the intricate cellular and molecular mechanisms underlying engineering cell approaches.

## 8. Conclusions and Future Directions

Osteosarcoma majorly develops in adolescence, causing a dramatic decrease in living quality. The reason for its poor prognosis and therapeutic challenges is due to immune escape. In addition, OS cells can invade other organs and develop resistance towards therapeutics. The above activity is closely associated with the TME; thus, a comprehensive understanding of the complicated OS-TME is significant. In the first part of this review, we discussed the roles of crucial immune cells in the OS-TME including OS-Ms, OS-Ns, MDSCs, and Treg. Given the importance of the TME, remodeling the OS-TME has surfaced as a key strategy to treat OS. Therefore, we highlighted the use of nanotechnology to cover the regimens to modulate the OS-TME. Indeed, compared with the past, investigations into cancer cell communication with immune cells have transformed from a less-traveled road to a flow of traffic. Because of in-depth study, strategies focused on the interactions of cells have made successful progress. However, there are obstacles in this wide road.

Firstly, the heterogeneity of cells within the tumor microenvironment poses a significant challenge to the effectiveness of therapeutic approaches. This diversity is particularly evident in MDSCs and OS-Ms [214], even though both polymorphonuclear MDSCs (PMN-MDSC) and monocytic MDSCs (M-MDSCs) promote OS progression [215]. The heterogeneity of cells requires close attention when choosing therapeutics. For instance, the STAT3 less-expressed M-MDSCs can differentiate to anti-tumor DCs, and this complete elimination may cause decreased DCs in the OS-TME, leading to non-beneficial effects of OS treatment [109]. TAM has a distinct tumor-promoting sub-phenotype. It is reported that M2b is beneficial for anti-tumor activity, demonstrating the difficulty in choosing strategies. As a result, sophisticated platforms like “organ-on-chip” models may be necessary for more comprehensive assays and to gain a deeper understanding of the role MDSCs play in the context of cancer. Secondly, there is a gap between laboratory models and humans post-challenge in real-Sworld conditions. MDSCs in humans exhibit distinct characteristics when compared to their murine counterparts [214]. Specifically, human MDSCs are typically characterized by a lack or low expression of HLA-DR, a major histocompatibility complex class II receptor, and a high expression of CD33, a myeloid cell marker [216]. Enhancing understanding of this difference is indeed a critical issue that needs to be addressed to achieve expected therapeutic efficacy.
